# Diet and Male Fertility: The Impact of Nutrients and Antioxidants on Sperm Energetic Metabolism

**DOI:** 10.3390/ijms23052542

**Published:** 2022-02-25

**Authors:** Alessandra Ferramosca, Vincenzo Zara

**Affiliations:** Department of Biological and Environmental Sciences and Technologies, University of Salento, I-73100 Lecce, Italy; vincenzo.zara@unisalento.it

**Keywords:** spermatozoa, infertility, obesity, fatty acids, sugar, bioactive molecules, mitochondria, metabolism

## Abstract

Diet might affect male reproductive potential, but the biochemical mechanisms involved in the modulation of sperm quality remain poorly understood. While a Western diet is considered a risk factor for male infertility, the Mediterranean diet seems to protect against male infertility; moreover, the role of a vegetarian habitus in the preservation of sperm quality is controversial. The aim of this review is to analyze the molecular effects of single nutrients on sperm quality, focusing on their involvement in biochemical mechanisms related to sperm bioenergetics. It appears that diets rich in saturated fatty acids (SFA) and low in polyunsaturated fatty acids (PUFA) negatively affect sperm quality, whereas unsaturated fatty acids supplementation ameliorates sperm quality. In fact, the administration of PUFA, especially omega-3 PUFA, determined an increase in mitochondrial energetic metabolism and a reduction in oxidative damage. Carbohydrates and proteins are also nutritional modulators of oxidative stress and testosterone levels, which are strictly linked to sperm mitochondrial function, a key element for sperm quality. Moreover, many dietary natural polyphenols differentially affect (positively or negatively) the mitochondrial function, depending on their concentration. We believe that an understanding of the biochemical mechanisms responsible for sperm quality will lead to more targeted and effective therapeutics for male infertility.

## 1. Introduction

Nutrition can affect, negatively or positively, sperm quality [1,2,3], and this effect depends on both quantitative and qualitative aspects of a diet, such as the calorie content of each macronutrient (carbohydrates, proteins, and fats), as well as on the specific fatty acid, carbohydrate, and protein profiles. 

In this context, unhealthy hypercaloric diets and excessive intake of saturated and trans fatty acids have a negative impact on sperm quality and, therefore, on the fertilization process [4,5,6,7,8,9,10,11]. On the other hand, healthy dietary models are clearly associated with a better sperm quality, suggesting that nutritional interventions could have a key role in the preservation of male fertility [3,12,13,14]. Moreover, an adequate intake of antioxidant molecules has been quite effective in the prevention and/or in the treatment of male infertility [15,16,17].

Despite a solid body of evidence showing the effects of nutrients and antioxidant molecules on male reproductive potential, there is little knowledge concerning the potential mechanisms involved in the modulation of sperm quality.

The aim of this review is to summarize the most recent evidence regarding the impact of nutrients and antioxidant molecules on sperm quality, with a particular focus on their involvement in biochemical mechanisms related to sperm bioenergetics. 

We believe that an understanding of the biochemical mechanisms responsible for sperm quality will lead to more targeted and effective therapeutics for male infertility. 

## 2. Diets and Male Fertility

There is increasing evidence that dietary behavior is associated with semen quality parameters [18]. In recent decades, the main dietary pattern has become the so-called “Western diet”, which is the consequence of the “westernization” of human lifestyle. This diet is characterized by a high intake of industrially processed foods, rich in animal proteins, simple carbohydrates, trans and saturated fats, and poor in dietary fiber and essential unsaturated fatty acids. Recent studies have linked a Western-pattern diet to an increased risk of metabolic diseases, atherosclerosis, neurodegeneration, cancer, as well as infertility [6].

Differently from the Western diet, the Mediterranean diet, which is one of the healthiest dietary patterns, has evident health benefits [19], including benefits in terms of semen quality parameters [20,21,22]. This diet is characterized by a high consumption of legumes, cereals, fruits, vegetables, a moderate consumption of fish and wine, and a low consumption of dairy products and meat; olive oil is the main source of added fat. Thus, the Mediterranean diet is rich in monounsaturated fatty acids (MUFA), fiber, and antioxidants and low in saturated fat (SFA). 

Another dietary model, the vegetarian diet, is similar in dietary composition to the Mediterranean diet, but it does not include meat and meat products, poultry, seafood, and flesh from any other animal. Recently, there has been considerable interest in the impact of this diet on male fertility, because it has been proposed that this dietary model decreased semen quality [23,24,25].

Western, Mediterranean, and vegetarian diets are the most investigated dietary models in the field of nutrition and male reproduction. Therefore, in the following paragraphs, we will try to discuss the effects of various combinations of nutrients that are the main components of these dietary patterns on sperm quality and metabolism.

### 2.1. Western Diet as a Risk Factor for Male Infertility 

The current western dietary habits generally imply high sugar and high fat consumption, with the consequent intake of unbalanced diets and/or of an excess of calories [26]. Over the last several decades, the Western diet has been therefore a significant contributor to the growing rate of obesity, which has had a significant effect on fertility [3] through changes in hormonal levels, sperm function, and gamete molecular composition. However, the molecular mechanisms responsible for the causal links between obesity and male infertility are not totally clear.

Obesity has been shown to disrupt various components of the hypothalamic–pituitary–gonadal axis, causing hypogonadism [27], which is mainly associated with reduced levels of testosterone and fewer spermatozoa [28].

Increased amounts of adipose tissue cause insulin resistance and have an important role in the development of oxidative stress, thus altering reproductive pathways and sperm function [29,30]. Hyperinsulinemia and hyperglycemia, which are the hallmarks of insulin resistance, seem to be responsible for the reduction in sperm glucose uptake and metabolism [31], thus having a possible role in the impairment of glycolysis in sperm cells. It is important to underline that glycolysis, along with oxidative phosphorylation (OXPHOS), is a metabolic pathway producing adenosine triphosphate (ATP), which is the primary source of energy for spermatozoa [32,33]. According to this hypothesis, diabetic male rats showed a decrease in sperm motility [34,35], which was restored after insulin administration [35]. 

Glucose uptake and homeostasis may be also modulated by leptin released from fat cells in adipose tissue depots. It has been found that hyperinsulinemia and hyperleptinemia were associated with an increase of insulin and leptin concentrations in seminal plasma, which may negatively impact male reproductive function and sperm quality [36].

Leptin has also an important role in the chronic pro-inflammatory state in the testicular microenvironment and/or excurrent ductal system, thus increasing the level of reactive oxygen species (ROS) which are responsible for the decrease in sperm quality [4,29,37]. 

Excess fat tissue results in the increased activity of aromatase, which is an enzyme responsible for converting testosterone to estradiol [38]. The consequent decrease in testosterone levels results in low sperm production, because this hormone is the major androgen in the testis involved in the regulation of spermatogenesis. Low testosterone levels seem to be related not only to oxidative stress but also to mitochondrial dysfunction in Leydig cells [39,40], located in the connective tissue surrounding the seminiferous tubules, where the first step of the synthesis of testosterone occurs.

Defects in Leydig cell mitochondria are responsible for oxidative damages in lipids, proteins, and mitochondrial DNA (mtDNA) and cause a decrease in ATP levels and an increase in ROS production [40]. Germ cells and mature spermatozoa are susceptible to oxidative stress, which leads to a decrease in sperm quality (decrease in sperm number and motility and increase in abnormalities in sperm morphology) [41,42,43]. 

Different findings suggest that sperm mitochondria play a pivotal role in the decrease of sperm quality caused by ROS. In germ cells, mitochondrial proteins and membrane lipids are damaged, and mtDNA is fragmented. Therefore, ATP synthesis is severely affected, and the decrease in energy production results in meiotic arrest, causing the presence of abnormalities in sperm morphology. High ROS levels also disrupt mitochondrial membranes and induce apoptosis, thus leading to a decrease in sperm number [40]. At the same time, in mature spermatozoa, the mitochondria are the target of ROS, and the decrease in mitochondrial functionality might be one of the causes responsible for the reduction of sperm motility [41].

It has been suggested that also dyslipidemia, which is known to be associated with increased amounts of adipose tissue, may have an impact on semen quality. In this regard, lipid profile alterations have been correlated with male infertility [4,5,42,43,44]. Dyslipidemia is a term referring to a group of different blood lipid imbalances such as hypercholesterolemia, hypertriglyceridemia, decrease of HDL–cholesterol, or combined hyperlipidemia. 

**Figure 1:**
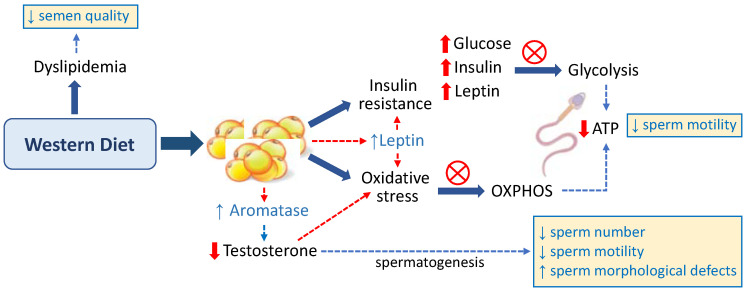
Effects of the Western diet on sperm quality.

The possible effects of the Western diet on sperm quality are schematized in Figure 1. 

### 2.2. Mediterranean Diet as a Protection Factor against Male Infertility

The Mediterranean diet incorporates the traditional healthy living habits of people from countries surrounding the Mediterranean Sea, although differentiated by some food choices and cooking practices. This dietary pattern has been shown to confer multiple health benefits. Interestingly, it seems able to promote good male reproductive health, being associated with an increase in sperm number and quality and with improved chances of conceiving [13,20,45].

One reason why the Mediterranean dietary pattern is so positive for male fertility is because it provides a low level of SFA and trans fatty acids and adequate levels of certain nutrients such as omega-3 fatty acids, antioxidant molecules, and vitamins. In fact, it has been shown that the intake of antioxidant vitamins and carotenoids was related to higher sperm counts [46]. Moreover, higher intakes of fruit, cereals, and vegetables were positively related to sperm motility and concentration [47,48].

Many dietary natural compounds isolated from fruits, vegetables, and edible plants can target the mitochondria, modulating their metabolism, biogenesis, and redox status [49,50,51]. The protection of mitochondrial function by these compounds may be important in explaining their beneficial effects on male reproductive performance [16].

Another characteristic of the Mediterranean diet is the consumption of olive oil as the main source of fat. It has been demonstrated in animal models that olive oil supplementation significantly increased sperm quality [5,42]. This is because olive oil, which is the main source of MUFA, may modify the sperm membrane lipid composition, reducing oxidative stress damages and restoring mitochondrial function [5].

Moreover, the Mediterranean pattern induces a reduction in omega-6 fatty acids in favor of omega-3 fatty acids, which have been associated with an improvement of sperm energetic metabolism [5]. 

### 2.3. Vegetarian Diet as a Controversial Factor for Male Infertility 

A substantial proportion of the world’s population is vegetarian for cultural or ethical values, religious beliefs, environmental concerns, and health considerations.

Existing studies have reported mostly protective associations between a vegetarian pattern and risk factors for chronic diseases [52]. However, the role of a vegetarian diet in the preservation of sperm quality is controversial.

Vegetables and fruits are rich in antioxidant molecules, which can act as sperm ROS regulators by reducing sperm DNA damage and by increasing sperm motility and vitality. At the same time, it has been described that the vegetarian diet reduced sperm concentration and motility, but its effect on infertility was not thoroughly assessed [25]. This effect may be attributed to estrogenic compounds or chemical residues in the diet which had a negative effect on sperm parameters [16,25,53,54]. 

## 3. Nutrients Impacts on Molecular Aspects Related to Sperm Quality

The differential impacts of Western, Mediterranean, and Vegetarian diets on male fertility depend on the amount and quality of the nutrients introduced. In the following paragraphs, we will analyze the molecular effects of single nutrients on sperm quality, focusing on their involvement in biochemical mechanisms related to sperm bioenergetics.

### 3.1. Dietary Fats

The negative impact of lipid metabolism disorders on male fertility is now well known, but the underlying molecular mechanisms involved are not totally clear. Dietary fats can influence the lipid composition of sperm cells, having harmful or beneficial consequences on male reproductive potential [55].

#### 3.1.1. Fatty Acids 

In sperm cells, fatty acids are constituents of the gamete membrane as well as energy suppliers [56] and can derive from de novo synthesis or from dietary sources. 

It is known that 18-carbon chain omega-6 and omega-3 polyunsaturated fatty acids (PUFA) cannot be endogenously synthesized by humans and therefore must be obtained from food. Long-chain PUFA, such as arachidonic acid (ARA; C20:4 omega-6) and eicosapentanoic acid (EPA; C20:5 omega-3), can derive from exogenous and endogenous sources. Vegetable oils, seeds, and nuts are a source of the omega-6 PUFA linoleic acid (LA; C18:2 omega-6) and α-linolenic acid (ALA; C18:3 omega-3). Seafood is a source of EPA and docosahexaenoic acid (DHA; C22:6 omega-3), which are long-chain omega-3 PUFA. Meat and dairy are a source of ARA.

Diets rich in SFA and low in PUFA or with an unbalanced omega-6/omega-3 PUFA ratio negatively affected sperm quality, whereas dietary unsaturated fatty acid supplementation ameliorated sperm quality [57]. 

In humans, the dietary intake of saturated fat negatively correlated with total sperm count and concentration; conversely, the intake of omega-3 fatty acids showed a positive correlation with sperm quality. However, the role of omega-3 fatty acids in the improvement of sperm quality is not always accompanied by changes in the fatty acid profile of sperm [55]. Therefore, a role of fatty acids in the regulation of sperm metabolism has been proposed [4,5,7]. 

To investigate the role of dietary fatty acids on sperm dysfunction, researchers used the model of diet-induced obesity in rodents and rabbits. They found that PUFA can influence reproductive processes, directly or indirectly, through several mechanisms. For example, PUFA may promote the loss of body fat, hence preventing obesity and then the development of infertility, but they are also able to act on specific aspects linked to male fertility. In fact, these fatty acids are also components of sperm membrane, whose fluidity and dynamics are necessary to promote fertilization. They are also precursors for eicosanoids synthesis, which can modulate many key enzymes involved in steroid metabolism. 

**Figure 2:**
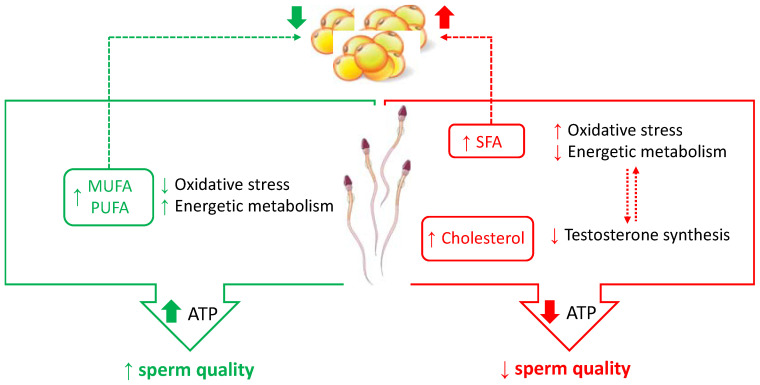
Effects of dietary fat on sperm quality. Dietary fatty acids exert a parallel modulation of lipid metabolism and sperm mitochondrial function. On the one hand, they may promote loss or increase of body fat, hence modulating molecular aspects related to obesity; on the other hand, they modulate oxidative stress and energetic metabolism. A dietary cholesterol excess could induce cholesterol accumulation in testicular Leydig cells, causing a decrease in testosterone production, which is related to oxidative stress mitochondrial dysfunction.

The molecular mechanisms responsible for the effects exerted by PUFA on male fertility may be due to a parallel modulation of lipid metabolism [58] and of sperm mitochondrial function [5] (Figure 2). Regarding this last aspect, a nutritional modulation of specific enzymes involved in sperm bioenergetics pathways has been proposed [5]. Among these enzymes, the sperm lactate dehydrogenase isoenzymatic form (LDH-C4 or LDH-X) is an important site of nutritional modulation by omega-3 PUFA [5]. This enzyme, which is present both in the mitochondrial matrix and in the cytosol of spermatozoa, with a net prevalence in the cytosol [59], catalyzes the conversion of pyruvate to lactate with the concomitant oxidation of NADH (reduced form of nicotinamide adenine dinucleotide) to NAD^+^ (oxidized form of nicotinamide adenine dinucleotide), playing a key role in the energy metabolism of spermatozoa [32,33]. In fact, LDH-C4 allows the parallel progress of glycolysis, by regenerating NAD^+^, and of OXPHOS, by the transport of reducing equivalents from the cytosol into the mitochondria. Increased activity of LDH-C4 after omega-3 PUFA administration was accompanied by a parallel increase in the activity of respiratory complexes. Moreover, dietary administration of omega-3 PUFA reduced oxidative damage on sperm cells, as also suggested by the increase in the aconitase/fumarase activity ratio [5]. Aconitase and fumarase are two Krebs cycle enzymes, and their activity ratio is used as an indicator of mitochondrial ROS production [60].

Interestingly, the activity of LDH-C4, pyruvate dehydrogenase, and respiratory enzymes was decreased after the administration of a diet rich in SFA and low in PUFA [4], thus suggesting that a specific fatty acid composition of the diet can counteract the negative effects of a high-fat diet on sperm cells. 

Recent studies demonstrated that also the administration of MUFA counteracted the negative effects of a hyperlipidic diet on sperm quality. The effects of MUFA were evaluated by adding olive oil or avocado extracts to experimental diets [5,42,61,62]. In fact, olive oil consists mainly of oleic acid (up to 83%), whereas oleic acid contributes to about 60% of the total fatty acid content of avocados.

The Authors found that MUFA supplementation modified sperm membrane composition, reduced oxidative stress damages, and modulated enzymatic activities involved in energetic metabolism [5,7,61] (Figure 2). 

#### 3.1.2. Dietary Cholesterol 

The study of the relationships between dietary cholesterol intake and male fertility is difficult to carry out in humans, and most of the information has been generated using rabbits as animal models. In fact, rabbits are sensitive to a cholesterol-enriched diet, showing a lipid metabolism which is closer to that of humans than to that of rodents [42,63,64,65,66,67]. 

It has been demonstrated that hypercholesterolemia modifies plasma membrane composition and dynamics, thus modifying sperm morphology and function [55]. Although rabbits are the gold standard for studying the relationships between hypercholesterolemia and male fertility, also data obtained in mice and rats suggested that an overload of dietary cholesterol has a very negative impact on male fertility [68,69,70].

The dietary cholesterol impact on male fertility is still poorly understood at the molecular level. What is known is that cholesterol is an essential lipid for membrane structure and dynamics, as well as for testosterone synthesis. In fact, the cholesterol levels in the sperm membrane affect membrane fluidity, which plays a key role in sperm motility, capacitation, and acrosome reaction [33]. 

At the same time, dietary cholesterol could induce cholesterol accumulation in testicular Leydig cells. Although cholesterol is the substrate for testosterone biosynthesis, an excess of cholesterol can be dangerous, because high cholesterol levels are responsible for the activation of the endoplasmic reticulum stress, causing the downregulation of steroidogenic enzymes and then a decreased testosterone production [71] (Figure 2). As reported before, low testosterone levels are strictly related to oxidative stress and to mitochondrial dysfunction (Figure 2).

Therefore, cholesterol concentration is critical to assure sperm quality. Cholesterol homeostasis is under the control of the transcription factors known as sterol regulatory element-binding proteins (SREBPs). It has recently been found that, in the short term, high circulating cholesterol levels due to diet decreased the expression of molecules involved in the cholesterol regulatory pathway, such as SREBP2 and its targets. In the long term, this short-term protective effect governed by SREBP2 became deregulated. The consequent increase in the membrane levels of cholesterol may be responsible for sperm abnormalities [72].

### 3.2. Dietary Carbohydrates 

The role of dietary carbohydrates on sperm quality is an aspect still largely unexplored. Sugar is present in almost all fruits and vegetables in the form of glucose and fructose, and a higher intake of fruits and vegetables is associated with improved semen parameters [2,20]. On the other hand, Chiu et al. found that sugar-sweetened beverage consumption was correlated with lower sperm motility in healthy young men [73]. On the other hand, reproductive hormone levels, as well as other sperm quality parameters, remained unaffected.

A link between sugar intake and lower sperm motility may be found in the increase of insulin resistance, which corresponds to a scarce utilization of glucose by sperm [31]. In sperm cells, glucose is the main substrate for glycolysis, where it is metabolized to pyruvate and/or lactate to obtain cellular energy in the form of ATP. Thus, s reduction in sperm glucose uptake and metabolism may correspond to a decrease in ATP concentration, which is necessary to sustain sperm motility. 

Increased blood glucose levels were also accompanied by a decrease in testosterone levels and an increase in oxidative stress [74]. Sperm mitochondria are a common target of oxidative stress and testosterone levels, which decreased their functionality. The reduced mitochondrial respiratory efficiency might be responsible for the decrease in sperm motility [41].

Very recently, the reduction in sperm motility observed after the administration of a high-sugar diet was also linked to an alteration of human sperm small RNA profiles [75]. In fact, mature sperm cells have a rich and diverse profile of small RNA, which is determined during the testicular phases of their development and displays a considerable plasticity in response to environmental insults [76,77]. Therefore, small RNA remodeling during post-testicular maturation of mammalian sperm has an essential role in the production of functionally mature spermatozoa [78].

Experiments carried out in rats suggested that a high fructose intake starting at juvenile age can impair the reproductive function. A high fructose intake lowered serum testosterone and sperm count. A possible effect on sperm motility was also proposed [79]. 

Although complex carbohydrates have been shown to offer multiple advantages in terms of overall health, there are no studies about their role in the preservation of male reproductive potential. 

Instead, some studies evaluated the effects on male fertility of artificial sweeteners, that have become increasingly popular as an alternative to sugar. In this context, some animal studies suggest that they may not be safer for male fertility than real sugar. For example, a recent study carried out in mice showed that high doses of aspartame correlated with sperm DNA fragmentation and morphologic defects. These effects were due to the increased production of reactive species, weakening the antioxidant defense system, and the consequent induction of oxidative stresses [80]. Stevia, the natural sweetener native to South America, was also associated with decreased sperm count and lowered testosterone level [81]. On the other hand, it was reported that the most common sucralose does not impair sperm quality and has no effect on sperm glycolysis [82].

### 3.3. Dietary Proteins 

Proteins are not energetic substrates for sperm cells [32,33]; moreover, a high-protein diet had no significant effect on glycemic control [83]. However, a low-protein diet has been considered a potential risk factor for male-factor infertility, causing a significant reduction in testis, epididymis, and seminal vesicle weights, as well as a decrease in serum testosterone [84]. Conversely, the reports on the effect of a high-protein diet present in the literature are contradictory [85].

Therefore, in addition to the level of dietary protein intake, also the type of protein is of significance, as amino acid profiles vary depending on the protein source. For example, plant-based proteins have lower sulfur-containing amino acids (methionine and cysteine) compared to animal proteins. Methionine, cysteine, and phenylalanine can affect sperm quality by decreasing their progressive motility in vitro [86]. 

A study conducted in monkeys evaluated the influence of animal and plant protein diets on sperm quality. When compared to monkeys receiving a plant-based diet, monkeys fed an animal protein diet showed lower sperm counts and motility and increased sperm abnormalities [87]. 

### 3.4. General Aspects concerning Caloric Nutrients and Sperm Metabolism 

A wide spectrum of exogenous factors, including caloric nutrients, affect sperm quality and function by acting on sperm energetic metabolism. In fact, since sperm rely on energetic substrates from their microenvironment to fuel their metabolism, it is easy to envision that all temporal changes in nutrient flux are directly reflected in sperm metabolism.

As reported in Table 1, oxidative stress and testosterone levels are the main players of this nutritional modulation and are strictly linked. In fact, low testosterone levels could be the result of mitochondrial defects caused by an excess of ROS in Leydig cells, where testosterone is synthesized. At the same time, a decrease in testosterone level could be a physiological response to reduced oxidative stress, since ROS are produced during steroidogenesis itself.

Gamete mitochondria are a common target of oxidative stress and testosterone levels and an important source of ROS. These organelles play a key role in sperm functionality [32,88,89] and can be considered a hub of cellular events related to energy production, ROS homeostasis, and steroid hormone biosynthesis. Therefore, all molecules that can influence this crosstalk may affect male fertility by targeting gamete mitochondria [16,54,90].

## 4. Antioxidants Impacts on Molecular Aspects Related to Sperm Quality

It has been shown that many dietary natural polyphenols (mainly flavonoids) isolated from fruits, vegetables, and edible plants modulate mitochondrial metabolism and biogenesis, as well as ROS homeostasis [49,50,51]. The modulation of mitochondrial function by these plant bioactive molecules may be important for the improvement of male reproductive performance.

In this context, it is important to underline that the mean polyphenol intake in the European population of 0.5–0.8 mg/day corresponds to polyphenol plasma concentrations of about 10 nM. For vegetarians and vegans, the mean polyphenol intake is 22.4 mg/day, and polyphenol plasma concentrations were estimated to be over 200 nM [91].

Quercetin is a dietary-derived bioflavonoid widely distributed in plants and vegetables, which has attracted considerable attention in the field of the study of male fertility owing to its potent antioxidant properties. However, controversial reports exist in the literature highlighting the antioxidant as well as the prooxidant properties of this flavonoid, leading to the “quercetin paradox in male reproductive dysfunction” [92]. The conflicting biological effects may be explained by a biphasic concentration-dependent response of sperm cells to quercetin. It has been recently demonstrated that quercetin stimulated the active state of mitochondrial respiration at concentrations of 0.1–1000 nM, also causing the uncoupling between electron transport and ATP synthesis in a dose-dependent manner starting from concentrations of 10 nM [16]. At the molecular level, quercetin interacts directly with mitochondrial membranes at the coenzyme Q-binding site, suppressing superoxide generation and stimulating the production of ATP [93,94,95]. At higher concentrations, quercetin can interact with lipid bilayers and membrane proteins, influencing the electric properties of mitochondrial membranes and uncoupling mitochondrial respiration from ATP synthesis [96]. 

Resveratrol is one of the most investigated natural polyphenolic compounds contained in several (more than 70) types of plants and in red wine. Some studies reported that resveratrol improves semen quality in humans, acting as a regulator of male reproductive function [97,98]. The effects of resveratrol on mitochondrial function have been investigated in different experimental models, and it has been demonstrated that it possesses antioxidant properties at low concentrations, while at high concentrations, its pro-oxidant properties could be responsible for detrimental effects on sperm mitochondria [16,99]. According to this hypothesis, it has been recently demonstrated that, starting from a concentration of 10 nM, resveratrol significantly uncoupled mitochondrial oxidative phosphorylation [16]. The investigation of the molecular mechanisms showed that resveratrol acts on mitochondrial metabolism via a sirtuin-dependent mechanism [50,75]. In this scenario, the positive effects of resveratrol on the control of mitochondrial metabolism have also been shown in metabolic disorders such as diabetes [100]. 

An hormetic effect on sperm quality has also been observed for naringenin, a flavanone commonly available in tomatoes, bergamot, and citrus fruits, that received some attention in the field of male reproduction for its antioxidant properties. Other molecules of plant origin that have been studied for their effects on human spermatozoa are apigenin, luteolin, and genistein. All these molecules, as well as quercetin and resveratrol, have also been recognized to display estrogenic activity and are also commonly known as phytoestrogens. Therefore, as multi-functional endocrine disruptors, they interfere with the enzymes needed for steroid biosynthesis and/or degradation [101]. This is an interesting aspect, since soy and soy-derived products, which have become more widely adopted by some vegan and vegetarian diets, contain isoflavones that mimic the actions of estrogens and may exert adverse effects on male fertility [102,103].

Among plant antioxidant molecules, lycopene is a lipophilic reddish carotenoid frequently found in tomatoes and several red fruits, with known antioxidant and free-radical scavenging activities [90]. It has been demonstrated that lycopene has positive effects on testicular mitochondrial function since it is a modulator of lipid peroxidation, antioxidant enzyme activities, and activity of the Krebs cycle [104]. This evidence suggests that lycopene may ameliorate mitochondrial respiration efficiency, and then sperm quality, in situations in which increased mitochondrial membrane lipid peroxidation might also contribute to impair mitochondrial functionality.

Astaxanthin is a xanthophyll carotenoid present in various microorganisms and marine organisms, with strong antioxidant properties, since it is 100-fold to 500-fold more effective than vitamin E in preventing lipid peroxidation [50,105]. A positive effect of astaxanthin on sperm parameters and fertility has been proposed [106,107,108], whose molecular basis can be explained by the improvement of mitochondrial function. In fact, astaxanthin appears to be able to increase mitochondrial membrane potential and respiratory control [109], which are important measures of mitochondrial functionality. 

The main antioxidant vitamins that help regulate free radicals in male reproduction are Vitamin E and Vitamin C [90,110]. Vitamin E is not synthesized by mammalians and includes a group of lipid-soluble compounds—tocopherols and tocotrienols—with alpha-tocopherol being the most active form. Vitamin E is found in plant-based oils, nuts, seeds, fruits, and vegetables and acts as an antioxidant, defending the organism against oxidative stress, thus having an important role in the protection of sperm membranes against ROS and lipid peroxidation. Therefore, it has been suggested that Vitamin E improves mitochondria activity, decreasing the damage to sperm and mitochondria [90,111]. 

Differently from Vitamin E, Vitamin C (ascorbic acid) is a very potent water-soluble antioxidant molecule, which has also a role in vitamin E recycle. It is found in many fresh fruits like oranges, lemons, limes, grapefruit, cantaloupes, mangoes, papayas, and their juices. Various studies have been carried out on the effects of ascorbic acid supplementation on sperm function, showing that the administration of vitamin C improves seminal quality [112,113,114]. The mechanism responsible for this effect involves a reduction of oxidative stress and an improvement of the antioxidant status, which change the microenvironment of the testes and enhance the production of energy needed for sperm motility. Molecular targets of Vitamin C are two mitochondrial enzymes, succinate dehydrogenase and ATPase [115].

## 5. Conclusions

Diet may be an important modifiable determinant of male reproductive potential. Therefore, the role of daily nutrient exposure needs to be highlighted to preserve male fertility or to prevent male infertility. A strong adherence to a healthy dietary pattern based mainly on plant foods and fish is positively correlated with indicators of sperm quality. 

Although the picture of the complex relationship between nutrients and sperm quality is far from complete, some indications can be drawn. First, the amount and quality of the nutrients introduced can affect sperm quality by acting on sperm energetic metabolism. Then, diets rich in SFA and low in PUFA or with an unbalanced omega-6/omega-3 PUFA ratio negatively affect sperm quality, whereas dietary unsaturated fatty acid supplementation ameliorates sperm quality. While an excess of simple carbohydrates negatively affects sperm function, there are no studies about the role of complex carbohydrates on male reproductive potential. Lastly, a low-protein diet, as well as the deficiency of some specific amino acids have been considered a potential risk factor for male-factor infertility. 

Fats, carbohydrates, and proteins affect sperm quality by acting on oxidative stress and testosterone levels, whose common target are the mitochondria. The mitochondria are key organelle supporting several sperm functions. Since they are involved in energy production, ROS homeostasis, and steroid hormone biosynthesis, all molecules that can influence their functions may affect male fertility. Among these molecules, dietary fatty acids and natural polyphenols act as modulators of sperm mitochondrial function.

**Figure 3:**
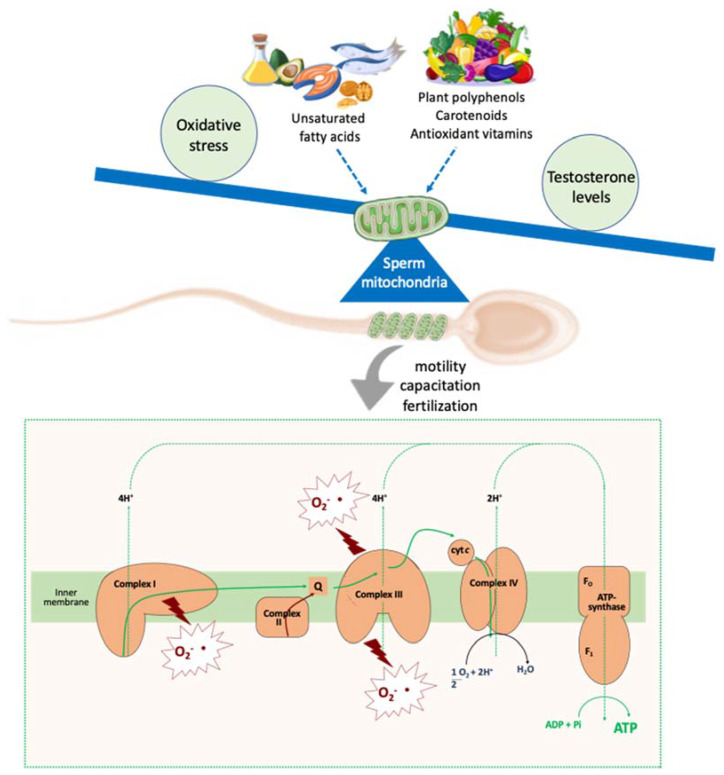
Dietary modulators of sperm mitochondrial function. Nutrients are modulators of oxidative stress and testosterone levels, which are strictly linked to sperm mitochondrial function, a key element related to sperm quality. In fact, in addition to their basic role in ATP synthesis, mitochondria are a major source of ROS, which are key mediators of cellular physiology and pathology. Fatty acids and several plant antioxidant molecules can target the mitochondria, improving and/or restoring their function, by acting on the ATP and ROS levels. These molecules can regulate sperm motility, capacitation, and fertilization, thus affecting sperm quality.

In fact, the administration of PUFA, especially omega-3 PUFA, determined an increase in the activities of mitochondrial enzymes involved in gamete energetic metabolism and a reduction in oxidative damage. Moreover, many dietary natural polyphenols (mainly flavonoids) found in fruits and vegetables differentially affect (positively or negatively) the mitochondrial function, depending on their concentration. Therefore, the modulation of sperm mitochondrial function could play a key role in the improvement of sperm quality (Figure 3). 

We are aware that our analysis provides only a small contribution to the field of nutrition and male reproduction. However, due to the importance of the role of diet on male infertility, whose frequency is increasing exponentially today, we believe that further investigation of the molecular mechanisms underlying the action of nutrients and natural compounds is necessary to develop new dietary approaches to preserve male reproductive potential.

## Figures and Tables

**Table 1:** Effects of nutrients on sperm quality.

	Sperm Quality	Molecular Mechanism	References
Cholesterol	↓	↓ membrane fluidity ↓ testosterone synthesis ↑ oxidative stress	[55,71]
SFA	↓	↑ insulin resistance ↓ sperm mitochondrial function ↑ oxidative stress	[4]
MUFA	↑	↑ membrane fluidity ↑ sperm mitochondrial function ↓ oxidative stress	[5,7,61]
PUFA	↑	↓ insulin resistance ↓ lipogenesis ↑ membrane fluidity ↑ sperm mitochondrial function ↓ oxidative stress	[5,57]
Carbohydrates	↓ (high sugar intake)	↑ insulin resistance ↓ testosterone synthesis ↑ oxidative stress small RNA profiles	[74,75,79]
Proteins	↓ (low protein intake)	↓ testosterone synthesis	[84]
